# Probabilistic Computing via Gate‐Tunable Random Telegraph Noise

**DOI:** 10.1002/advs.77554

**Published:** 2026-09-06

**Authors:** Gyungwon Yun, Jin Pyo Kang, Ho‐Sung Lee, Ik‐Hyeon Jeon, Ki‐Ju Park, Won‐Ju Cho, Seung‐heon Chris Baek, Joo‐Hyung Chae, Hamin Park

**Affiliations:** ^1^ Department of Electronic Engineering Kwangwoon University Seoul Republic of Korea; ^2^ Department of Electronics and Communications Engineering Kwangwoon University Seoul Republic of Korea; ^3^ Department of Electronic Materials Engineering Kwangwoon University Seoul Republic of Korea; ^4^ Center For Semiconductor Technology Korea Institute of Science and Technology Seoul Republic of Korea

**Keywords:** cmos compatible, electron trapping, mosfet, noise, optimization algorithm, probabilistic logic, randomness

## Abstract

Although probabilistic computing offers a route to quantum‐inspired optimization at room temperature, existing probabilistic bits (p‐bits) often depend on non‐CMOS devices or circuit‐intensive randomness. Here, we report a CMOS‐compatible p‐bit that harnesses intrinsic random telegraph noise (RTN) in a fully depleted silicon‐on‐insulator field‐effect transistor to produce stochastic binary outputs with direct gate‐voltage control. The capture and emission dynamics follow Shockley–Read–Hall (SRH) kinetics, yielding a quantitative sigmoid bias‐to‐probability transfer, and the dwell‐time statistics exhibit Poisson behavior consistent with memoryless switching. Pairwise statistical independence between independently measured RTN streams is supported through probabilistic Boolean operations and cross‐correlation analysis. Using the experimentally derived RTN probability response, we perform an RTN‐derived numerical emulation of simulated annealing for the two‐dimensional hydrophobic–polar protein‐folding benchmark, reaching low‐energy configurations with fewer stochastic update iterations than the number of candidate conformations evaluated by exhaustive enumeration. We further map the same update pipeline into a digital logic design using integer energy evaluation and lookup‐table probability mapping, providing a practical pathway toward CMOS‐based probabilistic accelerators. Collectively, these results elevate intrinsic transistor noise from an unwanted artifact to a gate‐tunable, CMOS‐compatible stochastic primitive, providing a physically grounded pathway toward CMOS‐based probabilistic processors.

## Graphical abstract

Intrinsic random telegraph noise in metal–oxide–semiconductor field‐effect transistors is harnessed to create gate‐tunable probabilistic bits. By modulating carrier‐trapping dynamics, the device produces stochastic binary outputs with a continuous bias‐to‐probability transfer. The experimentally derived response is then applied to energy‐based combinatorial optimization using a protein‐folding model, demonstrating a device‐informed pathway toward probabilistic computing.

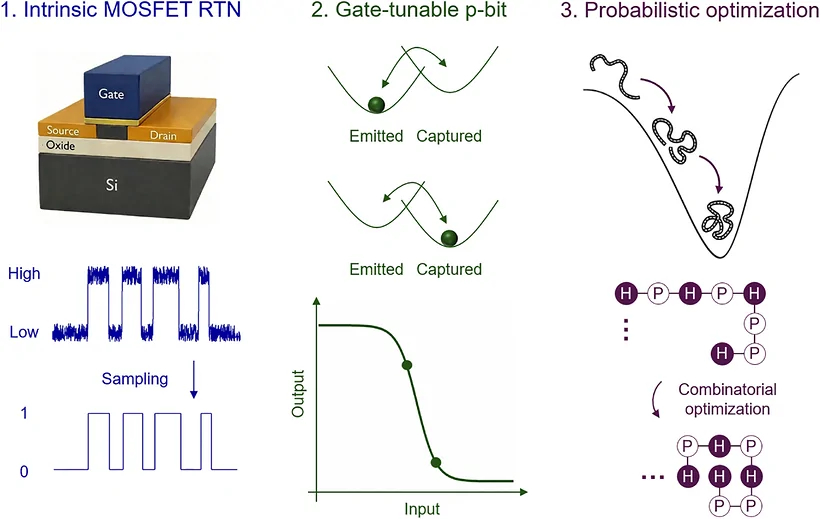

## 1 Introduction

As the computational demands of artificial intelligence [1], data‐centric workloads [2], and resource‐constrained edge devices [3] continue to increase, research interest in computing paradigms beyond conventional von Neumann architectures has intensified. Quantum computing is a prominent direction because quantum superposition can accelerate certain classes of complex operations; however, practical systems typically require cryogenic operation, which imposes substantial energy and system overhead [4, 5, 6]. These limitations motivate the search for alternative architectures that can perform complex computations efficiently under ambient conditions. To overcome these limitations, probabilistic computing (p‐computing) has emerged as an alternative paradigm that treats stochasticity as a computational resource. In p‐computing, information is encoded in probabilistic bits (p‐bits), which are stochastic units that continuously fluctuate between the binary states “0” and “1” [7]. The state occupancy is tuned by an input bias, enabling the hardware to sample configurations consistent with a Boltzmann distribution [8]. By enabling energy‐based probabilistic sampling, p‐computing provides a practical bridge between classical deterministic hardware and quantum‐inspired techniques such as quantum annealing. Recent studies have demonstrated p‐computing in diverse tasks, including combinatorial optimization [9], invertible Boolean logic [10], and Bayesian inference [11].

Previous demonstrations of probabilistic bits have followed two main routes. One uses fully digital or complementary metal–oxide–semiconductor (CMOS) heavy implementations, where probabilistic updates are realized through algorithmic randomness, lookup‐table‐based sigmoid approximations, and accept/reject comparisons driven by linear‐feedback shift register (LFSR)‐type pseudo‐random generators [12]. Although this approach is flexible, it inherently introduces substantial logic overhead and quantized probability representations, which can limit scalability and efficiency at the p‐bit level. The other route seeks the device‐level physical sources of stochasticity, including thermally activated switching in low‐barrier magnetic tunnel junctions (MTJs) [10], stochastic ion transport and filament dynamics in diffusive memristors [13], field‐induced nucleation variability in threshold‐switch oscillators [14], and impact‐ionized hole generation and recombination in biristor structures [7]. Recent studies have further broadened the hardware landscape to include CMOS transistor‐based and materials‐engineered platforms, including multi‐state probabilistic computing in floating‐body metal–oxide–semiconductor field‐effect transistors (MOSFETs) and in‐memory probabilistic computing in van der Waals ferroelectric devices with tunable probabilistic slopes and nonvolatile retention [15, 16]. At the architecture level, weighted p‐bits have been implemented on field‐programmable gate arrays using lookup table (LUT)‐based activation, digital pseudo‐random generation, and tiled p‐circuit structures for invertible logic and combinatorial problem solving [12]. Stochastic MTJ p‐bits have also been electrically coupled into asynchronous networks to experimentally perform probabilistic computation, including integer factorization [10]. More recently, sparse Ising machines have combined graph sparsification, graph coloring, and phase‐shifted updates of conditionally independent p‐bits to overcome the serial bottleneck of conventional Gibbs sampling and achieve massively parallel probabilistic sampling [17]. Together, these studies show that the development of larger p‐computing systems requires coordinated advances in stochastic‐bit generation, network connectivity, and update architecture.

Despite these advances, practical constraints remain in realizing efficient and robust probabilistic hardware. Emerging non‐CMOS devices often suffer from device‐to‐device nonuniformity and fabrication sensitivity, while some platforms exhibit slow stochastic dynamics with retention times spanning milliseconds. On the other hand, CMOS‐based implementations relying on impact‐ionization‐driven latch dynamics often require specific operating windows and relatively high voltages. These operating requirements can introduce trade‐offs in power efficiency, design simplicity, and long‐term device robustness. These considerations highlight the need for a CMOS‐native stochastic primitive that is compatible with conventional silicon transistor technology and offers gate‐tunable probabilistic behavior without specialized latch conditions or additional material integration.

Any device that exhibits controllable stochastic fluctuations under external bias can, in principle, function as a p‐bit. In this regard, random telegraph noise (RTN) in MOSFETs provides a native and well‐understood source of stochasticity. RTN arises from stochastic trapping and detrapping of carriers at defects in the gate oxide or at the oxide–semiconductor interface, producing discrete two‐level fluctuations in the drain current (I_D_) [18, 19, 20]. Because the capture and emission kinetics depend on gate bias through shifts in the trap energy (E_t_) relative to the channel Fermi level (E_F_), both the transition rates and state occupancy can be continuously tuned by the gate voltage (V_GS_) [21, 22]. From a probabilistic computing perspective, this enables a single transistor to act as a bias‐tunable probabilistic element at room temperature. Moreover, RTN can be realized within established CMOS process flows, offering a CMOS‐compatible pathway toward future integration of probabilistic and conventional circuits on the same chip.

In this work, we demonstrate room‐temperature probabilistic computing by elevating intrinsic MOSFET RTN from an unwanted noise source to a tunable, CMOS‐native stochastic primitive. We implemented a single‐transistor p‐bit whose binary state occupancy is directly tuned by V_GS_, providing a CMOS‐native, gate‐tunable physical entropy source for integration with larger digital or hybrid probabilistic‐computing architectures. We derived a quantitative mapping from trap capture and emission statistics to a sigmoid bias‐to‐probability transfer function based on Shockley–Read–Hall (SRH) kinetics, and we used this relation as a practical control law for probabilistic inference and optimization. To demonstrate end‐to‐end utility, we performed an RTN‐derived numerical emulation of the probabilistic update process and mapped the same probabilistic update pipeline into a hardware‐oriented register‐transfer level (RTL) design for a protein‐folding optimization task, showing how device‐level RTN characteristics can be translated into a system‐level probabilistic update rule and subsequently structured for digital hardware implementation. These results establish RTN as a physically grounded, CMOS‐compatible stochastic primitive for probabilistic computing that complements conventional deterministic logic.

## 2 Operating Principle of RTN‐Based p‐Bits

**FIGURE 1:**
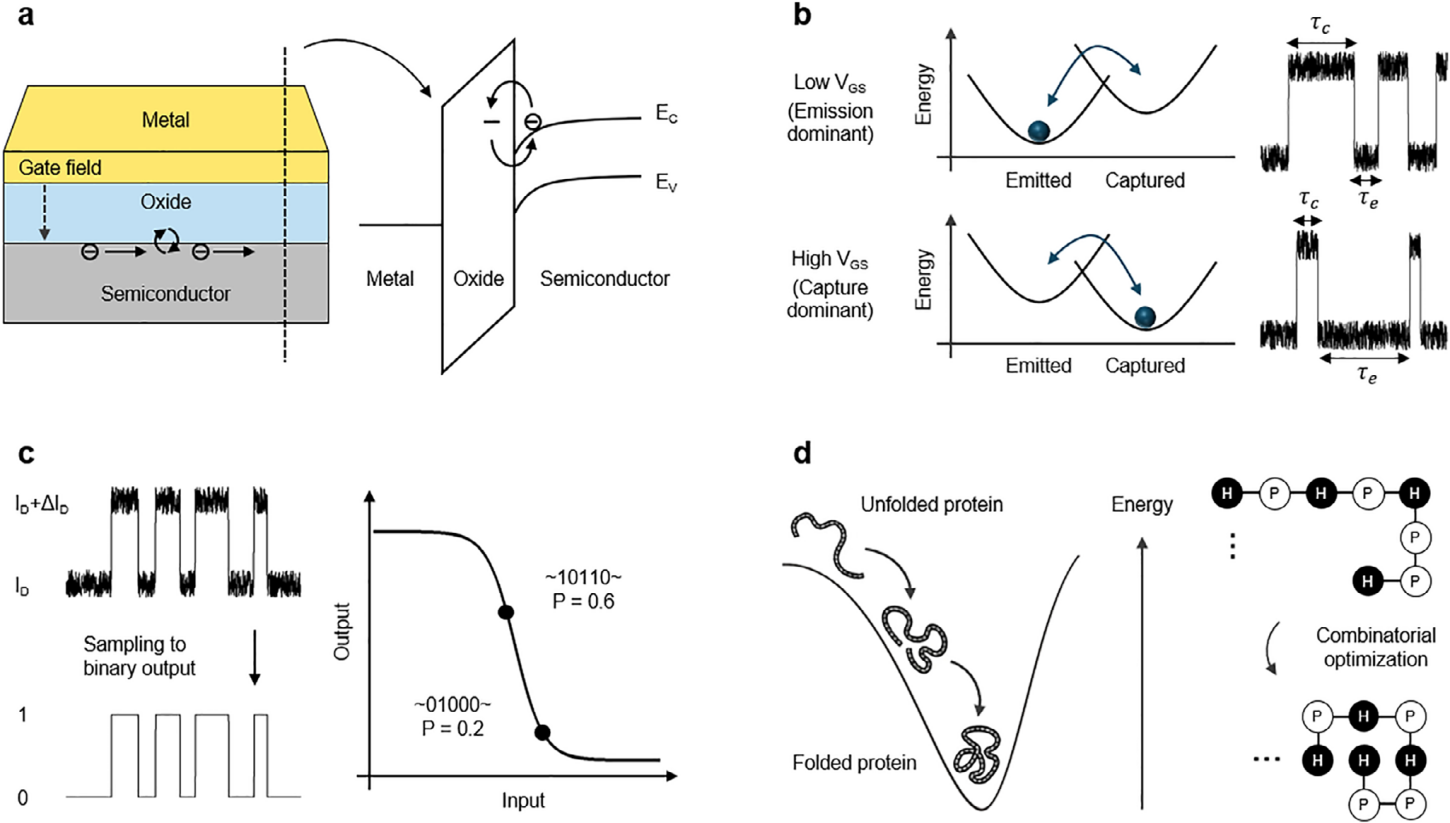
Operating principle of RTN‐based p‐bits and system‐level overview. (a) Schematic of carrier trapping and detrapping at an interface defect under a gate electric field, and the corresponding band‐diagram representation showing a trap level interacting with the semiconductor channel. (b) V_GS_‐dependent RTN kinetics. At low V_GS_, emission is dominant, whereas at high V_GS_, capture is dominant. The representative two‐level RTN waveforms are shown with characteristic capture and emission times, τ_c_ and τ_e_. (c) Construction of a p‐bit from RTN. The discrete drain‐current fluctuations between I_D_ and I_D_+∆I_D_ are sampled into a binary output, and the state probability is continuously tuned by the input bias, yielding a sigmoid‐like bias‐to‐probability transfer function. (d) System‐level demonstration. A p‐bit network performs energy‐based sampling to solve a protein‐folding optimization problem, illustrated by an energy landscape and a lattice hydrophobic–polar (HP) representation for combinatorial optimization.

Figure 1a–d show how a microscopic trapping event in an oxide defect can be translated into a controllable probabilistic bit and then elevated to a system‐level optimization engine. The main concept is to treat the intrinsic two‐level fluctuation associated with a single active trap as a native stochastic binary signal, and to use V_GS_ as a direct knob that tunes the state occupancy and switching statistics. RTN arises when charge carriers intermittently occupy and leave a localized defect state (trap) in the gate oxide or at the oxide–semiconductor interface (Figure 1a). Under an applied gate field, carriers can migrate between the channel and the trap. Each trapping or detrapping event perturbs the channel electrostatics and scattering environment, producing a discrete shift in the I_D_. Consequently, the current exhibits two stable levels separated by a characteristic amplitude ΔI_D_, corresponding to the two trap‐occupancy states.

The RTN waveform can be described by alternating residence times in the two current levels, as shown in Figure 1b. These residence times are commonly parameterized by the mean capture time (τ_c_) and mean emission time (τ_e_) [23]. Importantly, both τ_c_ and τ_e_ depend on V_GS_ because V_GS_ shifts the relative alignment between E_t_ and the channel carrier distribution. In the low‐V_GS_ regime, emission events tend to dominate, and the system spends more time in one current level, whereas in the high‐V_GS_ regime, capture events become more probable and the occupancy balance shifts. This gate dependence provides a practical handle to tune the fluctuation rate and the fraction of time the signal spends in each level. The ratio between τ_c_ and τ_e_ can be described by the SRH theory [24, 25], which yields the following relationship:

(1) $$\frac{\tau_{c}}{\tau_{e}}=exp\left(\frac{E_{T}-E_{F}}{k_{B}T}\right)$$

where k_B_ and T denote the Boltzmann constant and temperature, respectively. Capture and emission are transition processes rather than distinct probabilistic states. Therefore, we define the steady‐state probabilities in terms of the occupancies of the two RTN current levels. The empty (detrapped) state corresponds to the high‐current level and is assigned to logic “1,” whereas the occupied (captured) state corresponds to the low‐current level and is assigned to logic “0.” Here, τ_c_ denotes the mean residence time in the empty/high‐current state before a capture event occurs, and τ_e_ denotes the mean residence time in the occupied/low‐current state before an emission event occurs. Accordingly, the steady‐state probabilities are given by:

(2) $$P_{1}=P_{\mathrm{empty}}=P_{\mathrm{high}}=\frac{\tau_{c}}{\tau_{c}+\tau_{e}}={\left(1+\exp\left(\frac{E_{F}-E_{T}}{k_{B}T}\right)\right)}^{-1}$$

(3) $$P_{0}=P_{\mathrm{occu}\mathrm{pied}}=P_{\mathrm{low}}=\frac{\tau_{e}}{\tau_{c}+\tau_{e}}={\left(1+\exp\left(\frac{E_{T}-E_{F}}{k_{B}T}\right)\right)}^{-1}\;$$

where P_0_ + P_1_ = 1. The resulting state‐occupancy probability follows a Fermi–Dirac form. This sigmoid‐like response underpins our approach of exploiting RTN as a computational resource for probabilistic computing.

To construct a p‐bit, the two‐level I_D_ signal is sampled and converted into a binary output by assigning one level to logic “0” and the other to logic “1”. The resulting bitstream fluctuates stochastically, but its state occupancy is not fixed. Because the residence times in the two levels are set by τ_c_ and τ_e_, the steady‐state probability of observing either binary state is determined by their ratio. Because V_GS_ modulates the capture and emission statistics, it continuously tunes the output probability, producing a sigmoid‐like bias‐to‐probability transfer characteristic. In practice, this provides a direct and continuous “probability control law” that maps a V_GS_ input into the p‐bit output distribution. Once individual p‐bits provide bias‐tunable stochastic outputs, they can be coupled into networks that perform energy‐based sampling. The network dynamics explore configurations with a preference set via an effective energy function, enabling probabilistic inference and combinatorial optimization. As a representative example, we targeted protein‐folding optimization in a lattice hydrophobic–polar (HP) model, where the goal is to search a large configuration space for low‐energy folded states (Figure 1d). The conceptual bridge from device‐level stochasticity to system‐level computation is as follows: microscopic trap kinetics yield bias‐tunable randomness; this randomness is encoded as a controllable p‐bit, and coupled p‐bits enable optimization through energy‐landscape sampling.

## 3 Device‐Level Implementation of RTN‐Based p‐Bits

**FIGURE 2:**
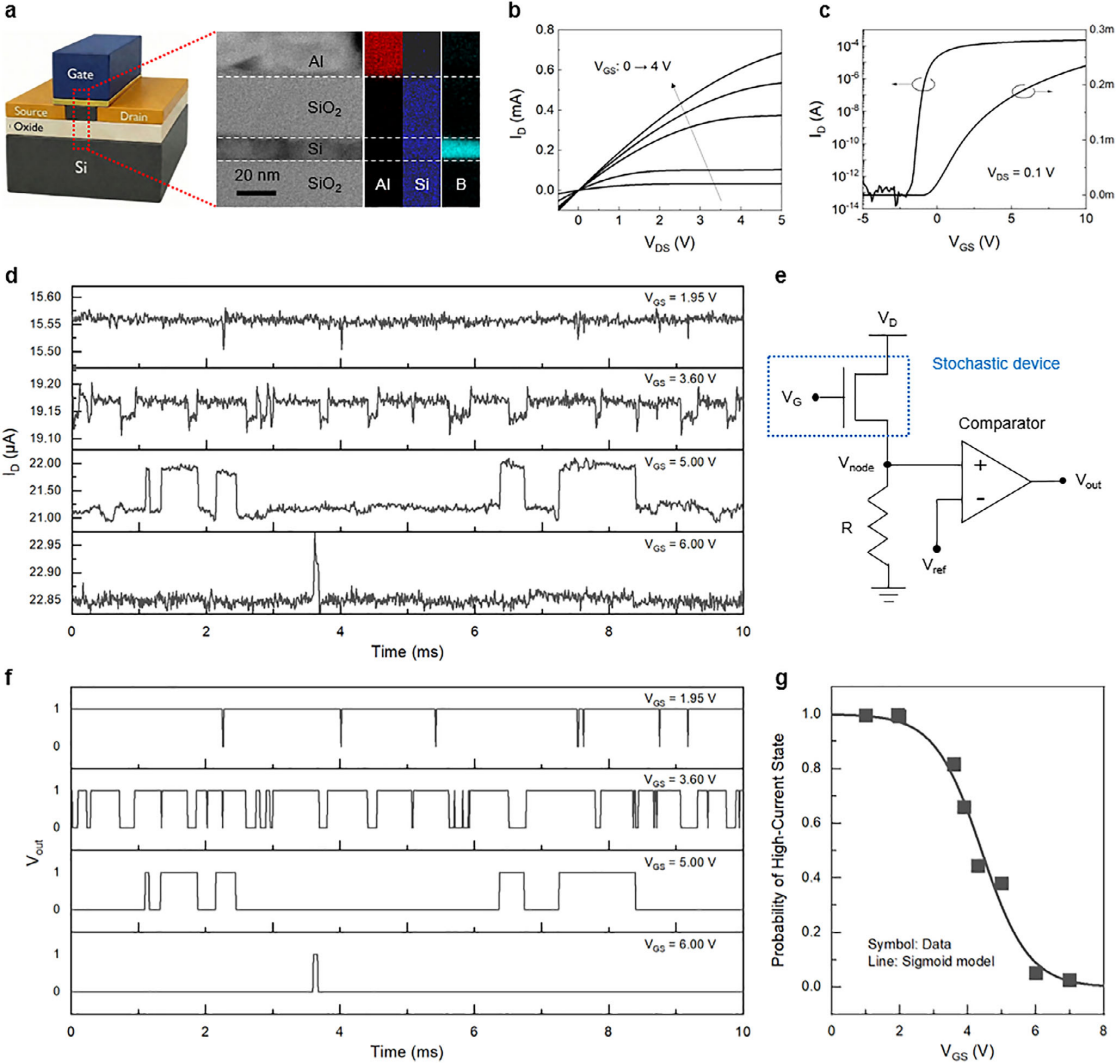
Device structure and RTN‐based p‐bit operation. (a) Schematic of the fabricated device and cross‐sectional structural/elemental analysis of the gate stack and channel region (Al/SiO_2_/Si). (b) Output characteristics (I_D_‐V_DS_) measured for V_GS_ values of 0–4 V. (c) Transfer characteristics (I_D_‐V_GS_) measured at V_DS_ of 0.1 V. (d) Time‐domain I_D_ traces showing two‐level RTN and its V_GS_ dependence for representative values. (e) Readout circuit used to convert the stochastic device output into a binary p‐bit signal. (f) Corresponding binary output waveforms (V_out_) obtained from the readout circuit demonstrating gate‐tunable state occupancy. (g) Measured probability of the high‐current (logic‐“1”) state as a function of V_GS_ (symbols), together with the fitted sigmoid model (line), confirming continuous bias‐to‐probability programmability.

To experimentally realize RTN‐based p‐bits, we fabricated a fully depleted silicon‐on‐insulator (SOI) transistor and verified its material stack and electrical characteristics. As shown in Figure 2a, the device layout and cross‐sectional analysis confirm a conventional metal, oxide, and silicon stack in which the gate field modulates carrier transport in the channel through the thin gate oxide. This structure provides a physically well‐defined environment for single‐defect activity, which is the microscopic origin of RTN. Figure 2b shows that the output curves exhibit a monotonic increase in I_D_ with V_DS_ for V_GS_ values of 0–4 V. The transfer curve measured at V_DS_ of 0.1 V shows a clear turn‐on with V_GS_, which defines a controllable operating window for subsequent time‐domain RTN characterization, as shown in Figure 2c.

Time‐domain I_D_ traces measured at representative values exhibit a discrete two‐level switching characteristic of RTN (Figure 2d). The fluctuation activity is strongly gate‐dependent: at low V_GS_, the current remains near a single level with rare switching events, whereas at intermediate V_GS_, frequent transitions occur, and at higher V_GS_, the waveform again becomes comparatively stable as the occupancy becomes biased toward one state. These gate‐controlled changes in both the transition rate and state occupancy provide the physical basis for probability programmability in the p‐bit. To convert the analog RTN signal into a binary stochastic output, we implemented the readout scheme shown in Figure 2e. The I_D_ is translated into a node voltage V_node_ across a load resistor R, and V_node_ is compared against a fixed reference V_ref_ to produce a digital output V_out_. V_ref_ was set to the midpoint between the two local signal levels identified before and after an abrupt RTN transition within a short sensing interval. This midpoint criterion maximizes the separation margin between the two states and reduces sensitivity to moderate threshold variation. The resulting binary waveforms are shown in Figure 2f. As V_GS_ is increased, the fraction of time that V_out_ stays in the “high” state changes continuously, demonstrating that the p‐bit probability can be directly programmed by the gate bias while retaining stochastic switching.

The gate‐tunable probability is quantified in Figure 2g as the time‐averaged occupancy of the high‐current, empty (detrapped), logic‐“1” state in the measured binary output. The probability varies smoothly with V_GS_ and is well described by a sigmoid dependence, which is consistent with the SRH‐kinetics‐based mapping between trap capture/emission statistics and the bias‐to‐probability transfer function. This experimentally calibrated sigmoid relation is used as the bias‐to‐probability control law for constructing and programming networks of RTN‐based p‐bits, as presented in the following sections. The several‐volt operating range demonstrated in the present device reflects the electrostatic design of its 50 nm SiO_2_ gate stack. Through engineering of the dielectric thickness, permittivity, and gate work function, the RTN probability‐control window can be adjusted toward lower‐ or higher‐voltage operation.

## 4 Gate‐Tunable RTN Kinetics and Stochastic Characteristics

**FIGURE 3:**
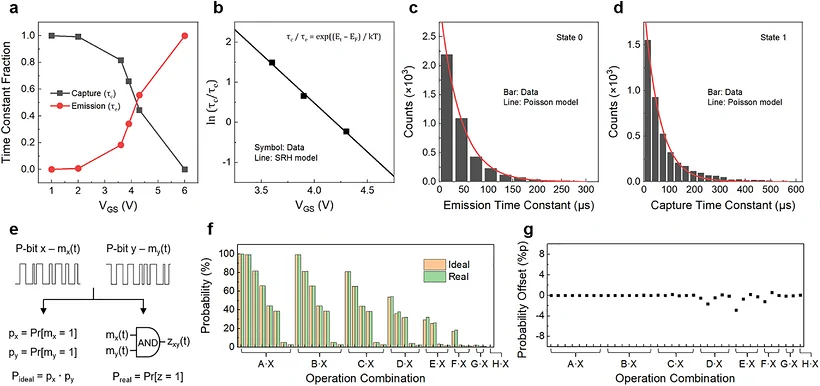
Gate‐tunable Shockley–Read–Hall (SRH) analysis of RTN kinetics with Poisson switching and independence verification. (a) Fractions of the extracted mean capture and emission times, τ_c_/(τ_c_+τ_e_) and τ_e_/(τ_c_+τ_e_), respectively, as a function of V_GS_, showing the gate‐dependent capture–emission balance. (b) Semi‐log plot of ln(τ_c_/τ_e_) versus V_GS_ with a SRH model fit, confirming SRH‐governed bias dependence of trap kinetics. (c) Histogram of emission‐time intervals for one RTN state with a Poisson (exponential) model fit. (d) Histogram of capture‐time intervals for the opposite RTN state with a Poisson (exponential) model fit. (e) Schematic of two p‐bits x and y and their Boolean (AND) operation, defining the ideal output probability (P_ideal_) and the experimentally obtained probability (P_real_). (f) Measured probabilities of logic outcomes for multiple operation combinations, comparing the ideal model and the experimentally obtained values. (g) Probability offset (P_real_ – P_ideal_) for each operation combination, quantifying the deviation from ideal probabilistic logic.

Figure 3a shows the normalized capture‐ and emission‐time fractions, defined as τ_c_/(τ_c_+τ_e_) and τ_e_/(τ_c_+τ_e_), respectively, extracted from time‐domain drain‐current RTN waveforms measured at different V_GS_ values. Both time‐constant fractions exhibit a strong gate dependence: as V_GS_ increases, the τ_c_ fraction decreases while the τ_e_ fraction increases.

This trend can be understood in terms of the electrostatic modulation of the channel surface potential by the gate bias, which shifts the E_F_ upward toward the conduction band and closer to E_t_. As voltage is applied to the gate metal, a potential difference forms across the gate oxide, thereby changing the channel surface potential and E_F_. Consequently, increasing V_GS_ reduces the energy difference (E_t_ – E_F_), raising the electron‐capture probability and shortening τ_c_, while suppressing emission and lengthening τ_e_. This physical illustration is further supported in Figure 3b, where ln(τ_c_/τ_e_) shows an approximately linear dependence on V_GS_, consistent with an SRH‐kinetics description. From the slope d[ln(τ_c_​/τ_e_​)]/dV_GS_, the effective trap location X_t_ inside the gate oxide was estimated using the nominal gate‐oxide thickness of T_ox_ = 50 nm. The extraction assumes a uniform electric field, corresponding to a linear potential drop across the SiO_2_ layer [26, 27]. Here, X_t_ is defined as the distance from the Si/SiO_2_ interface toward the gate electrode. Under these assumptions, the slope is expressed as:

(4) $$\frac{d\left(\ln\left(\tau_{c}/\tau_{e}\right)\right)}{dV_{GS}}=-\frac{qX_{t}}{k_{B}T\;T_{ox}}$$

Using Equation (4), we extracted an X_t_ value of 3.2 nm, indicating that the RTN‐active defect is electrically located within the gate oxide rather than arising from an external artifact. Because the extraction relies on the nominal oxide thickness and the assumed linear potential‐drop model, the 3.2 nm value should be regarded as an approximate effective electrical location rather than an exact structural coordinate. In addition, the extracted (E_t_ – E_F_) provides a quantitative handle on how the capture and emission balance evolves with V_GS_. At low V_GS_, E_t_ lies above E_F_, yielding a larger τ_c_/τ_e_, whereas at higher V_GS_, E_t_ approaches or falls below E_F_, increasing τ_e_ relative to τ_c_. This gate‐dependent evolution of the capture–emission balance produces a smooth transition between capture‐dominant and emission‐dominant regimes, which underpins the sigmoid‐like probability response used for p‐bit programmability.

To evaluate statistical independence, we randomly selected two RTN‐based p‐bits and performed a Boolean AND operation between them. As illustrated in Figure 3e, the two bitstreams m_x_(t) and m_y_(t) have individual probabilities; if they are independent, the probability that the AND output becomes “1” should follow P_ideal_ = p_x_ · p_y_. The measured “Real AND” probability P_real_ is obtained by bitwise AND at each time index and counting the fraction of ones in the resulting sequence. Figure 3f shows a comparison of P_real_ and P_ideal_ for multiple operation combinations, demonstrating close agreement over a wide probability range. The residual deviation is quantified (Figure 3g) by the probability offset (P_real_ – P_ideal_), which remains near zero for all tested combinations, supporting pairwise statistical independence between the two tested RTN streams under the measurement conditions.

Finally, the stochastic switching characteristics were further evaluated through dwell‐time, autocorrelation, cross‐correlation, and probability‐reproducibility analyses. As shown in Figure 3c,d, the histograms of the capture and emission dwell times are well described by exponential distributions, as expected for Poisson‐like switching kinetics [14, 28]:

(5) $$P\left(t\right)=\frac{1}{\tau }\;\exp\left(-\frac{t}{\tau }\right)$$

where t is the residence time in the corresponding RTN state, and τ is the associated mean residence‐time constant. The agreement with Equation (5) indicates that the state‐conditioned capture and emission kinetics are memoryless and Poisson‐like, such that the transition probability does not depend on the elapsed residence time [29]. In addition, the initial decays of the normalized autocorrelation functions were well described by exponential functions, yielding characteristic correlation times of 68 µs and 0.33 ms at V_GS_ = 3.60 and 5.00 V, respectively. The longer correlation time at 5.00 V is consistent with the longer characteristic residence times under this bias condition, further supporting gate‐dependent stochastic two‐state RTN switching.

Pairwise cross‐correlation analysis was also performed using five 10^4^‐point RTN segments at each gate‐bias condition. The mean zero‐lag cross‐correlation coefficient was 0.0075 at both V_GS_ = 3.60 and 5.00 V, while the corresponding mean absolute coefficients were 0.012 and 0.062, respectively. None of the ten segment pairs at either bias condition exhibited statistically significant correlation in an autocorrelation‐preserving circular‐shift test after Bonferroni correction, indicating no detectable reproducible time‐aligned linear correlation among the analyzed segments.

The reproducibility of the extracted gate‐programmed probability was further evaluated using correlation‐aware subsampling of the extended RTN sequences. Samples separated by at least five characteristic correlation times were partitioned into five disjoint, interleaved subsets. The resulting P_1_ values were 0.819±0.025 and 0.393±0.028 at V_GS_ = 3.60 and 5.00 V, respectively, where the variations represent the standard deviations among the five subsets. The gate‐induced difference in the mean probability was 0.426, more than 15 times larger than the maximum subset‐to‐subset standard deviation, supporting the reproducible extraction of the gate‐programmed probability despite the intrinsic temporal fluctuations of the RTN output.

These results demonstrate the operational reproducibility of the gate‐programmed probability over repeated stochastic switching events under the tested room‐temperature and fixed‐bias conditions. Because RTN originates from individual active defects whose spatial locations, energy levels, electrostatic coupling strengths, and capture and emission time constants are device‐specific, nominally identical devices may exhibit different RTN amplitudes, switching rates, usable gate‐bias windows, and P_1_(V_GS_) relations. Accordingly, device‐to‐device reproducibility should be understood as the preservation of gate‐tunable probabilistic functionality rather than exact matching of the sigmoid‐transfer parameters. These characteristics may also vary with temperature, prolonged bias stress, aging, oxide degradation, and operating history. A practical scaled implementation would therefore require device screening and initial and periodic calibration of both the readout threshold and the bias‐to‐probability transfer function. The readout threshold can be updated using a short pre‐sensing interval, while devices that do not exhibit resolvable two‐level RTN or a usable switching‐rate window can be managed through screening, remapping, or redundancy. Systematic assessment and improvement of usable‐device yield would further require process‐ and geometry‐controlled device arrays together with automated high‐throughput time‐domain screening.

The SRH‐consistent gate tunability, exponential dwell‐time distributions, decaying autocorrelation, absence of statistically significant inter‐segment cross‐correlation, reproducible probability extraction, and AND‐based joint‐probability test support the use of RTN as an intrinsic hardware stochastic source for gate‐tunable p‐bit operation and probabilistic computing.

## 5 RTN‐Based Probabilistic Optimization and Hardware Mapping

**FIGURE 4:**
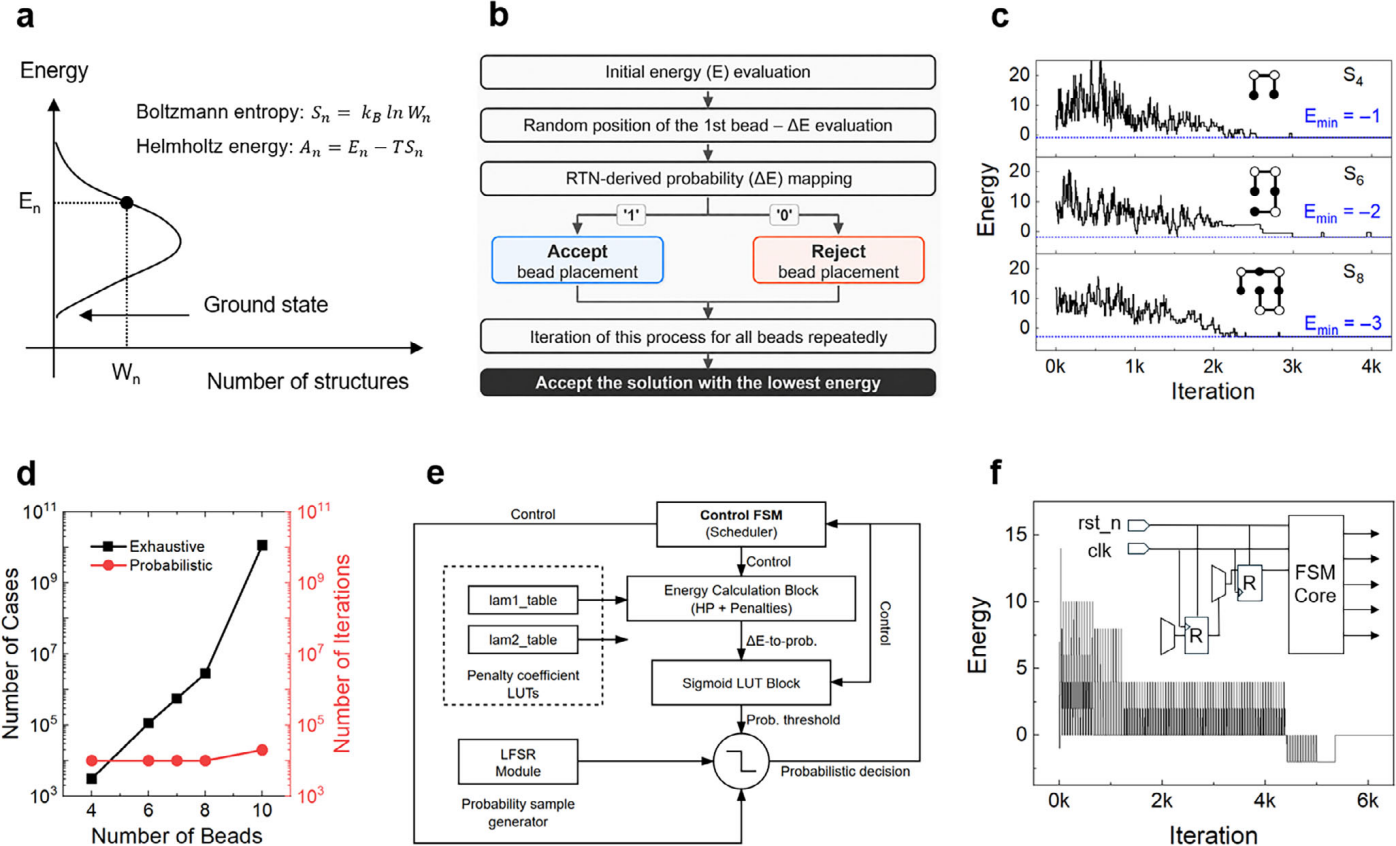
RTN‐derived probabilistic optimization, search‐space comparison, and hardware‐oriented mapping. (a) Thermodynamic view of the optimization landscape relating energy to configurational degeneracy and indicating the ground state as the target solution. (b) Flow chart of the RTN‐derived probabilistic search procedure in which ΔE is mapped to a probabilistic decision signal. (c) Representative energy trajectories versus iteration for different HP sequences, showing progressive convergence toward lower‐energy states. (d) Comparison versus problem size between exhaustive enumeration (black), expressed as the number of candidate conformations evaluated, and the probabilistic approach (red), expressed as the number of iterations required to reach the target low‐energy solution. (e) Top‐level hardware‐oriented architecture of the probabilistic solver. (f) Example energy‐versus‐iteration trace from the RTL‐mapped implementation, demonstrating stochastic descent and convergence toward a low‐energy regime; the inset shows the corresponding RTL control and datapath organization.

Protein folding can be interpreted as a search for the most stable configuration that minimizes the system's free energy, and the size of the conformational space grows exponentially with sequence length, rendering the problem NP‐hard [30, 31, 32, 33]. To capture the essential physics while keeping the formulation tractable, we used the two‐dimensional HP lattice model, in which each residue is classified as hydrophobic (H) or polar (P) and placed on a lattice. Because hydrophobic residues tend to cluster to reduce exposure, configurations that maximize H–H contacts correspond to the lowest‐energy (ground‐state) structures. This energy‐minimization view naturally aligns with a statistical‐mechanics interpretation in which lower‐energy configurations are exponentially more probable. This thermodynamic perspective is summarized in Figure 4a. The Boltzmann entropy is defined by the number of accessible structures W_n_ as S_n_ = k_B_lnW_n_, and the Helmholtz free energy can be written as A_n_ = E_n_−TS_n_ [34] (see Note S1 for a detailed thermodynamic framework). As the energy decreases, the degeneracy (number of structures) typically decreases, and the ground state is realized at the lowest energy with very limited degeneracy. This energy–entropy trade‐off motivates energy‐based probabilistic sampling as a strategy for exploring the folding landscape without explicit enumeration of all configurations.

We numerically emulated a probabilistic update rule based on the experimentally derived RTN bias‐to‐probability relation within a simulated‐annealing framework (see Note S2 for the complete cost‐function formulation and annealing schedule). Starting from an initial configuration, a trial move is generated by randomly selecting a bead position, and the corresponding energy change ΔE is evaluated. The calculated ΔE is mapped to an effective input value, denoted as V_in_ in Figure 4b, and the corresponding acceptance probability is obtained from the fitted RTN‐derived sigmoid relation. A numerical pseudo‐random number generator then provides a uniformly distributed sample for the accept/reject decision. Therefore, this numerical demonstration reproduces the experimentally established probability response of the RTN p‐bit but does not directly replay the measured RTN bit streams. In this way, energetically unfavorable moves may still be accepted with a finite probability, enabling escape from local minima and promoting global exploration. This accept/reject process is repeated sequentially across all beads and iterated, while tracking the best (lowest‐energy) configuration encountered.

Five HP sequences were considered in the numerical benchmark: S_4_ = HPPH, S_6_ = HPPHPH, S_7_ = PHPPHPH, S_8_ = HPHPHPPH, and S_10_ = HPPHPPHPPH, where the subscript denotes the number of beads. Figure 4c shows representative convergence behaviors for S_4_, S_6_, and S_8_, where the energy trajectories fluctuate as the algorithm explores competing configurations but progressively reach lower minima and eventually converge to the corresponding ground‐state energies. For S_8_ = HPHPHPPH, 100 independent simulations with different random seeds and initial conformations yielded a 73% success probability. Among the successful runs, the mean number of iterations to convergence was 5,478.4±1,630.3 (mean ± standard deviation).

Figure 4d compares, for S_4_, S_6_, S_7_, S_8_, and S_10_, the number of candidate conformations evaluated by exhaustive enumeration with the number of stochastic update iterations required by the RTN‐derived numerical emulation to reach the target low‐energy solution. Here, exhaustive enumeration refers to the evaluation of all candidate conformations under the same lattice constraints, rather than the runtime of a specific deterministic optimization algorithm. As the sequence length increases, the number of candidate conformations required for exhaustive enumeration grows rapidly, whereas the numerical emulation reaches the target low‐energy solutions with substantially fewer stochastic update iterations.

To examine a hardware‐oriented implementation pathway beyond the numerical demonstration, we mapped the same probabilistic folding pipeline into an RTL‐oriented digital implementation (the detailed system architecture and RTL module descriptions are provided in Note S3). Figure 4e presents the top‐level block diagram, which consists of (i) a main control unit that schedules bead updates, (ii) an energy‐compute engine that evaluates the HP interaction energy and constraint penalties, and (iii) a stochastic decision unit that performs ΔE‐to‐probability mapping and accept/reject decisions. Because real‐valued arithmetic and continuous exponential functions are expensive in digital hardware, the mapping adopts hardware‐friendly approximations, including integer‐based energy evaluation and a sigmoid‐approximation LUT to convert ΔE into an acceptance probability. In the present RTL implementation, the experimentally derived RTN bias‐to‐probability relation is realized through LUT‐based mapping, while an 8‐bit LFSR provides the digital sampling sequence for functional hardware verification. This RTL result demonstrates the hardware‐compatible organization of the device‐derived probabilistic update pipeline using integer energy evaluation, LUT‐based probability mapping, LFSR‐based digital sampling, and finite‐state machine (FSM)‐based control, providing a pathway toward future integration with a physical RTN source.

The envisioned implementation is a hybrid physical–digital architecture in which the digital circuitry performs energy evaluation and control, while a physical RTN p‐bit provides the stochastic output for the accept/reject decision. In this architecture, the calculated ΔE would determine the gate‐bias input of the RTN device, and the resulting comparator‐digitized stochastic output would replace the LUT‐ and LFSR‐based probability‐sampling path used in the present RTL implementation. Unlike a conventional LFSR‐based p‐bit, the physical RTN device intrinsically combines stochastic bit generation and gate‐tunable probability control within a single CMOS‐compatible transistor. Therefore, the device‐level and RTL‐level demonstrations establish complementary components of a future RTN‐integrated probabilistic accelerator. The RTL sequencing and datapath organization are detailed in the inset of Figure 4f. A start synchronization and pulse‐generation stage ensures deterministic triggering, a FSM core module orchestrates the iterative bead‐update process, and the energy datapath with update logic computes the current energy and updates a best‐energy register when an improved configuration is found. This structure implements the same loop shown in Figure 4b in a cycle‐accurate manner, while explicitly separating control flow, arithmetic datapath, and probabilistic decision logic. Figure 4f also shows an example energy‐versus‐iteration trace produced by the RTL‐mapped model in which the energy decreases in a stochastic, stepwise fashion and converges toward a low‐energy regime. Although integer quantization and LUT discretization can lead to differences from floating‐point numerical trajectories, the essential dynamics of probabilistic search, including energy fluctuations, gradual descent, and convergence, are preserved.

Overall, this hardware‐oriented mapping establishes an end‐to‐end pathway from RTN‐programmable stochasticity to a system‐level optimization task and its hardware‐oriented realization. The HP folding problem provides a concrete NP‐hard benchmark with a clear energy‐minimization objective, the experimentally derived RTN probability response enables probabilistic exploration of the energy landscape, and RTL mapping demonstrates that the same computation flow can be structured with LUT‐based probability approximation and FSM‐controlled datapaths, providing a hardware‐oriented pathway toward future RTN‐integrated probabilistic accelerators.

Table 1 presents a comparison of existing p‐bit device platforms, highlighting the key trade‐offs in entropy source, probability control, leakage, fluctuation speed, and CMOS integration. In most previous implementations, probability tuning is achieved indirectly through circuit‐level biasing or reference schemes, and the stochastic mechanism is not governed by a standard CMOS control terminal. In contrast, our device combines a physically well‐defined carrier trapping and detrapping entropy source with direct gate‐voltage programmability in a three‐terminal, CMOS‐native p‐bit that is compatible with standard CMOS processes. This architecture simultaneously maintains a clear off state with low leakage and a large on/off‐current ratio, while exhibiting experimentally validated kHz‐class stochastic fluctuations. These characteristics support intrinsic transistor noise as a single‐device, CMOS‐compatible physical stochastic source for probabilistic computing.

**TABLE 1:** Comparison of device platforms for p‐bit implementation.

	CMOS circuit	MTJ	Memristor	Threshold switch	Biristor	MOSFET
Entropy source	Pseudo‐random algorithm	Thermally activated magnetization switching	Cu‐ion filament formation and rupture	Field‐induced nuclei variability	Generation and recombination of impact‐ionized hole	Electron trapping and detrapping
Device structure	Complex (32‐bit LFSR + CMOS comparator/control logic)	Complex (Ta/Pt/[Co/Pt]_7_/…/MgO /CoFeB/Ta/Ru/Ta)	Metal/insulator/metal (Cu_0.1_Te_0.9_/HfO_2_/Pt)	Metal/insulator/metal (IMT(W/NbO_2_/W), OTS(W/C/STAG/W))	Emitter/base/collector (npn Si)	MOS (Al/SiO_2_/Si)
Device terminal	—	2	2	2	2	3
Probability control	Indirect (circuit‐based)	Indirect (V_ref_‐based)	Direct (V_in_‐based)	Indirect (V_comparator_‐based)	Direct (V_collector_‐based)	Direct (V_gate_‐based)
Off‐state device leakage		N/A (no true off‐state)	∼10^−12^ A	> 10^−6^ A (IMT) > 10^−9^ A (OTS)	∼10^−10^ A	∼10^−12^ A
On/off current ratio	—	< 2	∼10^5^	> 10^3^ (IMT) > 10^6^ (OTS)	∼10^6^	∼10^9^
Reported fluctuation/operation speed	100 MHz (base operating frequency)	1 kHz	1 kHz	8 MHz	1 kHz	3 kHz
CMOS compatibility	Full	Partial (non‐CMOS materials)	Partial (non‐CMOS materials)	Partial (non‐CMOS materials)	Full	Full
Reference	Ref. [12]	Ref. [10]	Ref. [13]	Ref. [14]	Ref. [7]	This work

Table note: The leakage current, on/off ratio, and fluctuation/operation speed are representative values reported under the device‐specific operating conditions of the cited studies. Because the definitions and measurement conditions differ across device platforms, these values are provided for qualitative comparison and are not directly normalized benchmarks.

## 6 Conclusions

We demonstrated a CMOS‐native probabilistic computing pathway that harnesses intrinsic current fluctuations in a fully depleted SOI transistor as a tunable entropy source for p‐bits. Gate‐bias modulation of carrier trapping and detrapping enables a single‐device stochastic output whose state occupancy is continuously tunable by V_GS_, and the resulting probability behavior is physically grounded through SRH‐based analysis of capture and emission kinetics, together with Poisson, memoryless switching statistics. Statistical independence between p‐bits is further supported by probabilistic Boolean operations that closely match ideal predictions with negligible correlation. Beyond device characterization, we demonstrated system‐level relevance by applying RTN‐derived probabilistic sampling to an NP‐hard protein‐folding task in the 2D HP lattice model, and we outlined a hardware‐oriented mapping of the same update pipeline using LUT‐based probability approximation and FSM‐controlled datapaths. Collectively, these results position intrinsic transistor noise not as a limitation but as a gate‐tunable, CMOS‐compatible physical stochastic source for probabilistic processors that complement conventional deterministic computing.

## 7 Methods

### 7.1 Device Fabrication

Fully depleted SOI MOSFETs were fabricated on SOI wafers with a lightly doped p‐type top silicon layer (thickness: 10 nm; BOX: 750 nm). The active channels (W/L = 4 µm/20 µm) were defined by photolithography and reactive ion etching. Source/drain contacts were formed using 100 nm n^+^ poly‐Si deposited using low‐pressure chemical vapor deposition. A 50 nm SiO_2_ gate oxide was deposited using RF sputtering (power/pressure: 200 W/4 mTorr), followed by e‐beam evaporation of an Al gate electrode (150 nm). Devices were subjected to forming‐gas annealing at 450°C for 30 min.

### 7.2 Electrical Characterization and RTN Measurement

The DC output and transfer characteristics were measured to confirm stable transistor operation before RTN acquisition. Time‐domain RTN traces were then recorded at room temperature using a Keysight B1500A equipped with a waveform generator/fast measurement unit (WGFMU) for high‐speed current sampling. RTN was acquired under a fixed V_DS_ of 0.1 V while varying the gate bias to modulate capture/emission kinetics. The acquisition duration varied depending on the measurement condition. The analyzed RTN traces were acquired at 100 kHz for at least 0.1 s, yielding at least 10^4^ samples per trace. Depending on V_GS_, the number of detected state transitions ranged from several tens to several thousand. The transition intervals were used to extract τ_c_ and τ_e_ and construct the dwell‐time histograms, whereas the output probability was calculated from the state occupancy over the full trace. To minimize environmental interference, we conducted the measurements using shielded cabling and a low‐impedance, earth‐referenced grounding configuration.

## Author Contributions

**Gyungwon Yun**: conceptualization, writing – original draft, data curation, investigation, validation, visualization, formal analysis, methodology. **Jin Pyo Kang**: writing – original draft, conceptualization, investigation, validation, visualization, formal analysis, data curation. **Ho‐Sung Lee**: software, data curation, validation, methodology. **Ik‐Hyeon Jeon**: software, data curation, validation, methodology. **Ki‐Ju Park**: methodology, resources, investigation. **Won‐Ju Cho**: methodology, investigation, resources. **Seung‐heon Chris Baek**: writing – review and editing, conceptualization, supervision, validation. **Joo‐Hyung Chae**: writing – review and editing, supervision, validation. **Hamin Park**: writing – review and editing, conceptualization, supervision, funding acquisition, validation, project administration.

## Code Availability Statement

The computer code used in this study is available from the corresponding authors upon reasonable request.

## Conflicts of Interest

The authors declare no conflicts of interest.

## Supporting information




**Supporting File**: advs77554‐sup‐0001‐SuppMat.docx.

## Data Availability

The data that support the findings of this study are available from the corresponding author upon reasonable request.
