# Supplementary material for: Probabilistic Computing via Gate‐Tunable Random Telegraph Noise
Source: Adv Sci (Weinh). 2026 Sep 6:e77554. Online ahead of print. doi: 10.1002/advs.77554 (PMC13547700; doi:10.1002/advs.77554)
Supplement: Supplementary file 1 [file ADVS-9999-e77554-s001.docx]

Supplementary Information for

Probabilistic Computing via Gate-Tunable Random Telegraph Noise

Gyungwon Yun^1^†, Jin Pyo Kang^1^†, Ho-Sung Lee^2^†, Ik-Hyeon Jeon^2^, Ki-Ju Park^3^, Won-Ju Cho^3^, Seung-heon Chris Baek^4,*^, Joo-Hyung Chae^2,*^, and Hamin Park^1,*^

^1^Department of Electronic Engineering, Kwangwoon University, Seoul 01897, Republic of Korea

^2^Department of Electronics and Communications Engineering, Kwangwoon University, Seoul 01897, Republic of Korea

^3^Department of Electronic Materials Engineering, Kwangwoon University, Seoul 01897, Republic of Korea

^4^Center for Semiconductor Technology, Korea Institute of Science and Technology, Seoul 02792, Republic of Korea

†These authors contributed equally to this work.

*Corresponding author. Email: seungheon.baek@kist.re.kr (S.-h. C. Baek), jhchae@kw.ac.kr (J.-H. Chae), parkhamin@kw.ac.kr (H. Park).

**Supplementary Note 1: Thermodynamic Framework of the HP Lattice Model**

The protein folding problem is modeled as a search for the global minimum in a thermodynamic energy landscape. In the 2D hydrophobic–polar (HP) model, the stability of a conformation is governed by the Helmholtz free energy (A), which balances the internal energy (E) and the entropy (S)^1^:

$A = E - TS$	(S1)

Entropy is defined by the Boltzmann entropy formula: S = k_B_ lnΩ, where Ω is the degeneracy, or the number of possible microstates (conformations) available at a specific energy level. High-energy states (unfolded) have high degeneracy (high entropy), whereas the native ground state (folded) has unique or near-unique degeneracy (low entropy). Energy is determined by the number of hydrophobic contacts.

The goal of the optimization is to minimize A. At high "temperatures" (in the context of simulated annealing), the TS term dominates, allowing the system to explore high-entropy states. As T decreases, the E term becomes dominant, driving the system into the low-energy ground state defined by the HP interactions.

**Supplementary Note 2: Energy-Based Network Representation and Cost Function Formulation**

**2.1 Collective Energy and Probabilistic Update Rule**

The collective energy of the network configuration m is defined as^2^:

$E_{m}(m)=-\sum_{i,j} J_{ij}m_{i}m_{j}-\sum_{i} h_{i}m_{i}$	(S2)

where $m_{i}\in\{0, 1\}$ denotes the instantaneous state of the i-th p-bit, W_ij_ represents the coupling strength between p-bits (derived from spatial constraints and hydrophobic interactions), and h_i_ represents local biases.

The system evolves via a heat-bath update rule. At each step, a p-bit flip (or a move in the lattice) is accepted with a probability determined by the change in energy (∆ E) and the current temperature (T):

$P(accept)=\left( 1+\exp\left( \Delta E/k_{B}T \right) \right)^{-1}$	(S3)

In the numerical emulation, this heat-bath acceptance probability is realized by mapping ΔE and the effective temperature to the experimentally fitted RTN sigmoid response. Through these stochastic updates governed by the prescribed acceptance probability, the ensemble of p-bits samples configurations according to the Boltzmann distribution:

$P(m)=\frac{1}{Z}\exp\left( -E_{m}(m)/k_{B}T \right)$	(S4)

where Z is the partition function. This stochastic acceptance allows the solver to escape local minima, naturally evolving toward low-energy states and providing a classical analog of annealing.

In the simulated-annealing update, we use an effective temperature parameter $T$ (not the physical ambient temperature) to control stochastic acceptance. $T$ is scheduled to decrease geometrically as $T_{k}=T_{0}(T_{F}/T_{0})^{k/STEPS}$, thereby gradually reducing the acceptance of uphill moves across iterations. The effective annealing temperature parameter was initialized at T_0_ = 10.0 and geometrically reduced to $T_{F}$ = 10⁻⁵ over the full iteration schedule. A relatively high initial temperature promotes exploration by allowing energetically unfavorable moves, whereas the final temperature effectively suppresses uphill acceptance, approaching near-deterministic behavior and stabilizing low-energy configurations.

**2.2 Protein Folding Cost Function Formulation**

The global energy landscape is mapped directly onto the p-bit network to describe the folding behavior of the HP protein model on a lattice. The total cost function (H_total_) is constructed as the sum of physically motivated energy terms, namely the hydrophobic contact energy (E_HP_), single-occupancy constraint (E_1_), and chain connectivity constraint (E_2_):

$H_{total}=E_{HP}+\sum_{i=1}^{2} \lambda_{i}E_{i}$	(S5)

where $\lambda_{i}$ denotes the Lagrange multipliers that determine the relative strength of each constraint term^3^.

**Hydrophobic Contact Energy (E_HP_):** This term drives the folding process by rewarding the clustering of hydrophobic residues.

$E_{HP}=-N_{HH}$	(S6)

where $N_{HH}$ is the number of hydrophobic-hydrophobic (H-H) pairs occupying adjacent lattice sites but not adjacent in the primary sequence. Because this term has a negative sign, each adjacent H-H contact reduces the total energy, thereby favoring compact structures^4^.

**Single-Occupancy Constraint (E_1_):** This ensures that each residue $f$ occupies exactly one lattice cell $s$.

$E_{1}= \sum_{f} \left( \sum_{s} \sigma_{f}^{s}-1 \right)^{2}+(same for odd parity)$	(S7)

where $\sigma_{f}^{s}\in\{0,1\}$ is a binary variable indicating whether residue $f$ is located at position $s$. A positive quadratic penalty is introduced if a residue is placed in multiple cells or none. Because the lattice is divided into even and odd parity sublattices to represent alternating residue positions, this condition is applied separately to each parity group.

**Chain Connectivity Constraint (E_2_):** This imposes the chain connectivity condition, ensuring that consecutive residues along the sequence occupy adjacent lattice sites.

$E_{2}= \sum_{1\leq f<N} \sum_{s} \sigma_{s}^{f} \sum_{\left\| s^{'}-s \right\|>1} \sigma_{s^{'}}^{f+1}$ + $\{same with odd even parity interchanged$} (S8)

For each pair of consecutive residues $(f, f+1)$, the term contributes a positive penalty proportional to $\lambda_{2}$ if they are separated by more than one lattice unit ($||s'-s|| > 1$). This prevents discontinuities in the chain. In our simulations and hardware mapping, large values of $\lambda_{1}$ and $\lambda_{2}$ were selected to make invalid configurations energetically prohibitive, effectively forcing the p-bit network into the subspace of valid physical structures.

**Supplementary Note 3: FPGA and RTL Hardware Implementation Details**

**3.1 System Architecture**

The probabilistic folding algorithm was mapped to a digital logic design using Verilog HDL and synthesized using the Xilinx Vivado Design Suite. The architecture is modular and consists of a central Finite State Machine (FSM), a Pseudo-Random Number Generator (PRNG), and arithmetic units for energy evaluation.

**3.2 Module Descriptions**

- **fpga_protein_folding_fsm (Control Unit):** It cycles through the states S_IDLE, S_INIT, S_PICK, S_E0, S_WAIT_CAND, S_E1, S_DECIDE, and S_DONE, corresponding to initialization, candidate selection, energy evaluation, and stochastic acceptance.
- **lfsr8 (Digital Pseudo-Random Sampling Source):** In the FPGA emulation, an 8-bit LFSR provides the pseudo-random threshold used for the stochastic accept/reject logic.
- **energy_calc (Integer Arithmetic):** To reduce hardware overhead, we converted the floating-point energy calculations to fixed-point integer arithmetic. The cost function H_total_ is calculated using simple adders and comparators rather than complex multipliers.
- **sigmoid_lut.mem (Probability Mapping):** The Fermi-Dirac activation function is implemented as a pre-computed lookup table (LUT) indexed by the integer energy difference (ΔE) and a temperature index. The LUT outputs an 8-bit acceptance threshold (p_accept), which is compared with a pseudo-random number to realize the stochastic accept/reject decision, thereby avoiding computationally expensive exponential operations on-chip.

**3.3 Constraints Handling in Hardware**

The penalty coefficients (λ_1_, λ_2_) are stored in ROM blocks (lam1_table.mem, lam2_table.mem). The energy_calc module accesses these values to apply immediate heavy penalties if a proposed move violates chain connectivity or overlaps, ensuring that the RTL-mapped implementation rapidly discards invalid geometries.

**References**

1	Brady, G. P. & Sharp, K. A. Entropy in protein folding and in protein—protein interactions. *Curr. Opin. Struc. Biol.* **7**, 215-221 (1997). <https://doi.org/10.1016/S0959-440X(97)80028-0>

2	Cipra, B. A. An Introduction to the Ising Model. *Am. Math. Mon.* **94**, 937-959 (1987). <https://doi.org/10.1080/00029890.1987.12000742>

3	Irbäck, A., Knuthson, L., Mohanty, S. & Peterson, C. Folding lattice proteins with quantum annealing. *Phys. Rev. Res.* **4**, 043013 (2022). <https://doi.org/10.1103/PhysRevResearch.4.043013>

4	Yoda, T., Sugita, Y. & Okamoto, Y. Hydrophobic Core Formation and Dehydration in Protein Folding Studied by Generalized-Ensemble Simulations. *Biophys. J.* **99**, 1637-1644 (2010). <https://doi.org/10.1016/j.bpj.2010.06.045>
